# Preoperative neutrophil-lymphocyte axis and postoperative mortality: an INSPIRE analysis

**DOI:** 10.1515/biol-2025-1378

**Published:** 2026-10-30

**Authors:** Ting Gong, Peitong Xue, Yifei Liao, Jiao Xu, Mingrong Tan, Haijin Wang, Weiwei Liu

**Affiliations:** Department of Anesthesiology, Xiangyang No.1 People’s Hospital, Hubei University of Medicine, Xiangyang, 441000, China; Department of Orthopedics, Suizhou Hospital, Hubei University of Medicine, Suizhou, 441300, China

**Keywords:** neutrophil-lymphocyte ratio (NLR), abdominal surgery, postoperative mortality, systemic inflammation, INSPIRE database

## Abstract

Systemic inflammation is linked to postoperative outcomes. This study evaluated the association between preoperative neutrophil-lymphocyte axis markers (neutrophil count, lymphocyte count, neutrophil-to-lymphocyte ratio [NLR], and lymphocyte-to-neutrophil ratio [LNR]) and all-cause mortality following abdominal surgery. We conducted a retrospective cohort study using the Informative Surgical Patient dataset for Innovative Research Environment (INSPIRE) database. Associations with mortality were assessed using Cox regression, with results reported as hazard ratios (HR). Survival disparities and dose-response relationships were evaluated via Kaplan-Meier analysis and restricted cubic splines, respectively. Subgroup analyses tested the robustness of findings. Among 7,165 patients (mean age 59.8 ± 13.6 years; 60.7 % male), 1,754 deaths (24.5 %) occurred. After adjustment, higher neutrophil count (HR = 1.03, 95 % CI 1.01–1.05, *P* = 0.001) and higher NLR (HR = 1.20, 95 % CI 1.11–1.30, *P* < 0.0001) were associated with increased mortality risk, while higher LNR was associated with lower mortality (HR = 0.67, 95 % CI 0.55–0.82, *P* < 0.0001). Lymphocyte count showed an inverse trend (HR = 0.93, 95 % CI 0.86–1.00, *P* = 0.05). Survival probabilities differed significantly across marker quartiles (all *P* < 0.0001). Dose-response analyses indicated non-linear relationships for LNR and lymphocyte count, but linear associations for NLR and neutrophil count. Subgroup analyses confirmed consistent associations without significant interactions. Preoperative neutrophil-lymphocyte axis markers independently predict mortality in abdominal surgery patients and may aid risk stratification and personalized management.

## 1 Introduction

Abdominal surgery is among the most frequently performed surgical procedures worldwide. Despite significant advancements in surgical techniques and perioperative care, postoperative mortality remains a critical and persistent clinical challenge, underscoring the need for improved risk stratification strategies [1], [2], [3]. The identification of reliable, easily obtainable preoperative prognostic markers is therefore essential for optimizing individual patient risk assessment, guiding clinical decision-making, and ultimately improving surgical outcomes.

The inflammatory response is a pivotal factor in the pathogenesis of postoperative complications and mortality [4]. A dysregulated immune response, characterized by an excessive or prolonged pro-inflammatory state, can contribute to organ dysfunction and failure. Neutrophils and lymphocytes are core cellular components of the innate and adaptive immune systems, respectively. Their absolute counts, as well as derived ratios, reflect the dynamic balance between pro-inflammatory and anti-inflammatory processes, offering a window into a patient’s immunological status. The neutrophil-to-lymphocyte ratio (NLR) and its inverse, the lymphocyte-to-neutrophil ratio (LNR), are simple, cost-effective inflammatory biomarkers derived from routine complete blood count (CBC) tests, requiring no additional resources [5], [6], [7].

Previous research has validated the prognostic utility of NLR and LNR in various surgical contexts, including oncologic, cardiac, and orthopedic surgery cohorts [8], [9], [10]. However, evidence regarding their association with mortality specifically following abdominal surgery remains inconsistent. Furthermore, there is limited data exploring the precise dose-response relationships between these hematological markers and mortality risk, as well as their prognostic performance across different patient subgroups (e.g., stratified by age, surgical urgency, or type of abdominal procedure). These gaps hinder the translation of these biomarkers into standardized clinical practice for abdominal surgical patients.

The INformative Surgical Patient dataset for Innovative Research Environment (INSPIRE) provides a valuable, large-scale, publicly accessible resource for perioperative medical research, featuring granular clinical, laboratory, and outcome data. Leveraging this robust database, this retrospective cohort study aimed to comprehensively investigate the association of preoperative neutrophil count, lymphocyte count, NLR, and LNR with all-cause mortality in a broad population of patients undergoing abdominal surgery. We hypothesized that these readily available hematological markers would serve as independent predictors of postoperative mortality. We further hypothesized that elucidating their non-linear dose-response relationships with mortality would provide nuanced clinical insights, enhance risk stratification models and inform targeted perioperative management strategies.

## 2 Materials and methods

### 2.1 Study population

This retrospective cohort study utilized data from the INSPIRE database, which includes approximately 130,000 patients who underwent surgical anesthesia at Seoul National University Hospital between 2011 and 2020. The INSPIRE dataset was approved by the Institutional Review Board (IRB) of Seoul National University Hospital (SNUH, IRB No. H-2210-078-1368) in compliance with the 1964 Declaration of Helsinki and its subsequent amendments. Due to the retrospective nature of the study, the IRB waived the requirement for informed consent. All data in the INSPIRE dataset are publicly available on the PhysioNet website (https://physionet.org/content/inspire/1.3/), and no additional ethical approval was required for the current analysis.

The inclusion criteria for this study were: (1) patients who underwent abdominal surgery based on the International Classification of Diseases, Tenth Revision (ICD-10) procedure codes (detailed in Supplementary Table S1); (2) availability of preoperative neutrophil count, lymphocyte count, and other relevant laboratory data; (3) availability of postoperative survival status data. The exclusion criteria were: (1) body mass index (BMI) less than 16 or greater than 40-as these extremes of malnutrition or severe obesity could independently alter the association between inflammatory markers and mortality; (2) multiple abdominal surgeries; and (3) missing data for other covariates.

**Ethical approval:** This retrospective cohort study used publicly available INSPIRE database. The original INSPIRE dataset was approved by the Institutional Review Board of Seoul National University Hospital (IRB No. H-2210-078-1368) and performed in accordance with the Declaration of Helsinki. No additional ethical approval was required for the present secondary analysis.

### 2.2 Exposure factors

The exposure factors were preoperative neutrophil count, lymphocyte count, NLR, and LNR. Neutrophil and lymphocyte counts were obtained from routine preoperative CBC tests. NLR was calculated as the ratio of absolute neutrophil count to absolute lymphocyte count, while LNR was computed as the inverse ratio. All laboratory measurements were obtained within 7 days prior to surgery, with the most recent measurement used for analysis.

### 2.3 Outcome measure

The primary outcome was all-cause mortality after surgery, which was further categorized into in-hospital mortality, 90-day mortality, 180-day mortality, 365-day mortality, and long-term mortality during follow-up. Survival time was defined as the interval from the date of surgery to death or the last follow-up. Follow-up data were extracted from the INSPIRE database.

Mortality data were collected from three sources: (1) hospital electronic medical records, (2) prospective follow-up records, and (3) the Korean National Death Registry. For deaths occurring after hospital discharge, standardized structured telephone interviews were conducted to confirm survival status, and the information was cross-validated with national registry data to ensure accuracy. All outcomes were recorded as binary variables (dead or alive). Patients without valid follow-up information were excluded. As this was a retrospective observational study, blinding was not feasible during outcome assessment; however, all-cause mortality is an objective hard endpoint that minimizes the risk of subjective bias.

### 2.4 Covariates

The selection of covariates was based on clinical significance, previous literature, and expert consensus. Detailed justification for the inclusion of each covariate is provided in Supplementary Table S5. Covariates adjusted in the regression models included demographic characteristics (age, sex, body mass index [BMI]), clinical variables (heart rate, body temperature, systolic blood pressure, diastolic blood pressure), laboratory parameters (creatinine, glucose, albumin, total protein, total bilirubin, hemoglobin), surgical variables (operative time), and comorbidities (hypertension, diabetes, chronic kidney disease [CKD], renal insufficiency, liver insufficiency). Additionally, the use of medications, including angiotensin-converting enzyme inhibitors (ACEI), angiotensin receptor blockers (ARB), and β-blockers, was adjusted for in the models. The American Society of Anesthesiologists (ASA) physical status classification was also incorporated as a covariate [11], [12], [13], with low-risk defined as an ASA score ≤ 2 and high-risk defined as an ASA score ≥ 3. Although detailed information on surgical indication (elective vs. emergency) and contamination class was not available in the INSPIRE database, we included ASA classification, operative time, and intraoperative hemodynamic variables as proxies for operative severity and urgency, to the extent possible given the data limitations.

### 2.5 Statistical analysis

Continuous variables were presented as mean ± standard deviation (SD) for normally distributed data or median (interquartile range [IQR]) for non-normally distributed data. Categorical variables were summarized as counts and percentages. Between-group differences were compared using the independent samples *t*-test, Mann-Whitney U test, or chi-square test as appropriate.

Cox proportional hazards regression models were used to evaluate the association between each exposure factor and mortality. Before constructing the models, we assessed multicollinearity among the covariates using the variance inflation factor (VIF), with a threshold of 10 indicating significant collinearity. All covariates had VIF values <5, confirming no significant multicollinearity, which satisfies the statistical assumptions for multivariable regression analysis and obviates the need to exclude any covariates (detailed VIF results are provided in Supplementary Table S4). Three models were constructed: Model 1 (unadjusted), Model 2 (adjusted for age, sex, and BMI), and Model 3 (fully adjusted for all covariates).

Because the neutrophil-to-lymphocyte ratio (NLR) and lymphocyte-to-neutrophil ratio (LNR) are mathematically reciprocal (LNR = 1/NLR), including both in the same regression model would introduce collinearity. Therefore, each marker was analyzed in separate Cox models. Both are reported to facilitate comparison with previous literature and to provide complementary clinical interpretations (higher NLR indicates a pro-inflammatory state, whereas higher LNR reflects a relatively better immune status). Each exposure factor was analyzed both as a continuous variable and as a categorical variable divided into quartiles (Q1–Q4) to explore dose-response relationships. The linear trend test was performed using the median value of each quartile, and the corresponding *P* value for trend was calculated.

Handling of missing data: The INSPIRE database contained missing values for some variables. Based on the data collection protocol and missing pattern analysis, the missingness was determined to be missing completely at random (MCAR). We adopted a complete case analysis approach: patients with missing data for any of the core variables (preoperative neutrophil count, lymphocyte count, or any covariate included in the fully adjusted model) were directly excluded from the analysis. No imputation methods (e.g., mean imputation, multiple imputation) were applied. To assess the potential impact of excluding incomplete cases on selection bias, we performed multiple robustness checks, including sequential adjustment Cox regression (Models 1–3), Kaplan-Meier survival analysis, restricted cubic spline analysis, and subgroup analyses. The consistency of findings across these complementary approaches supports the robustness of our results.

KM survival analysis was conducted to compare survival probabilities across quartiles of each exposure factor, with the log-rank test used to assess significance. RCS analysis was performed to visualize the dose-response relationships between each exposure factor and mortality, with knots placed at the 5th, 27.5th, 50th, 72.5th, and 95th percentiles. The likelihood ratio test was employed to evaluate non-linearity.

Subgroup analyses were conducted based on sex (female, male), age (<60 years, 60–80 years, >80 years), BMI (<18.5 kg/m^2^, 18.5–24 kg/m^2^, >24 kg/m^2^), hypertension (yes, no), and diabetes (yes, no). Interaction terms between each exposure factor and subgroup variables were included in the Cox regression models, and *P* values for interaction were calculated to assess the stability of the associations.

All statistical analyses were performed using R (version 4.5.0) software A two-tailed *P* value < 0.05 was considered statistically significant.

## 3 Results

### 3.1 Baseline characteristics

After excluding 647 patients with a BMI greater than 40 or less than 16 (as these extremes of severe obesity or malnutrition could independently alter the association between inflammatory markers and mortality), a further 7,853 patients (44.7 %) were excluded due to missing values for preoperative neutrophil count, lymphocyte count, or other covariates, and 1,887 patients were excluded due to multiple abdominal surgeries. Ultimately, 7,165 patients were included in the final analysis (Figure 1). The mean age of the cohort was 59.83 ± 13.62 years, and 4,347 (60.67 %) were male. The median operative time was 210.00 (130.00, 290.00) minutes. During follow-up, 1,754 (24.5 %) patients died. The median follow-up time was 11.00 (IQR: 9.00–43.00) months. Detailed information on missing data patterns and a comparison of baseline characteristics between included and excluded patients are provided in Supplementary Table S2. The number and percentage of missing values for each key variable are summarized in Supplementary Table S3.

**Figure 1:**
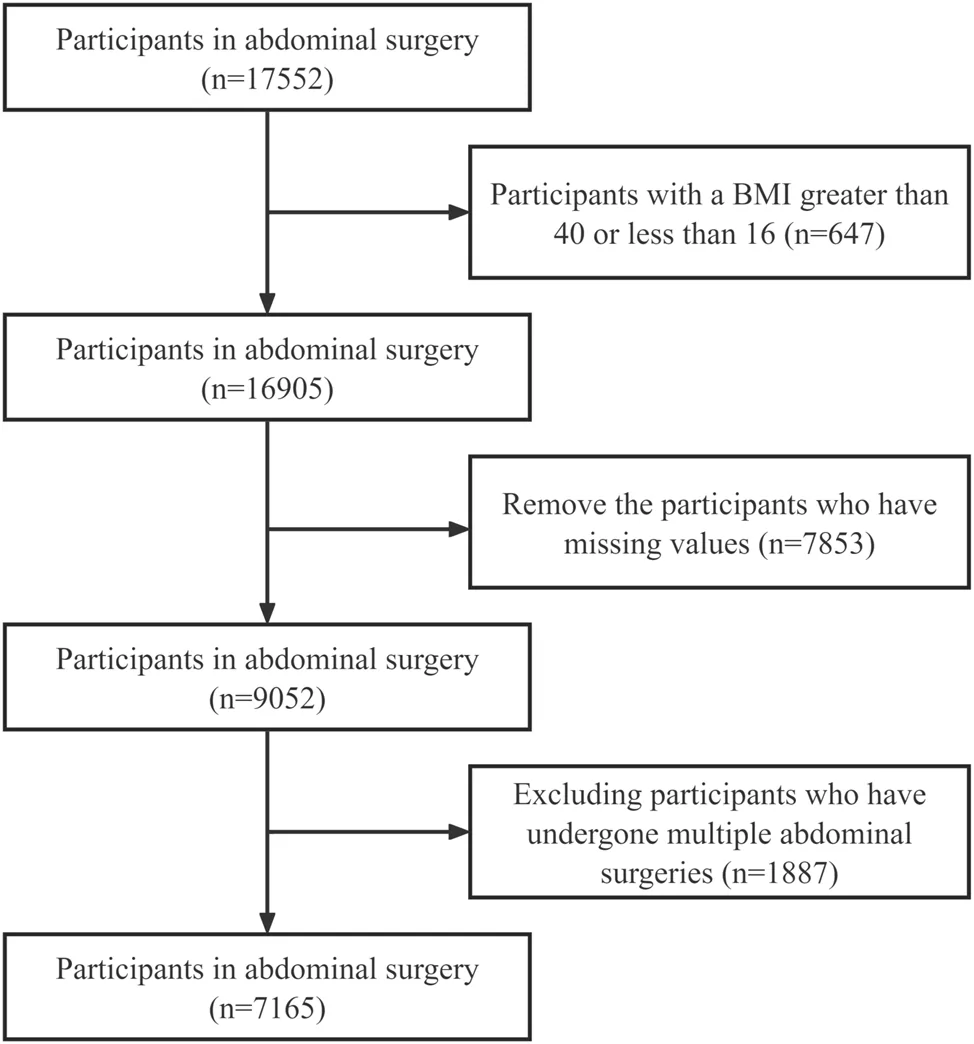
Screening flowchart.

Compared with survivors, non-survivors were older (66.05 ± 11.69 vs. 57.82 ± 13.60 years, *P* < 0.0001), had lower BMI (22.06 ± 3.36 vs. 23.64 ± 3.32 kg/m^2^, *P* < 0.0001), higher heart rate (76.51 ± 12.47 vs. 71.19 ± 11.04 beats/min, *P* < 0.0001), lower albumin levels (3.72 ± 0.51 vs. 4.06 ± 0.43 g/dL, *P* < 0.0001), lower total protein levels (6.65 ± 0.68 vs. 6.90 ± 0.58 g/dL, *P* < 0.0001), longer operative time (220.00 [140.00, 300.00] vs. 210.00 [130.00, 285.00] min, *P* < 0.0001), lower hemoglobin levels (12.20 [10.70, 13.70] vs. 13.20 [11.90, 14.20] g/dL, *P* < 0.0001), higher neutrophil count (4.40 ± 2.43 vs. 3.75 ± 1.74 × 10^9^/L, *P* < 0.0001), lower lymphocyte count (1.62 ± 0.71 vs. 1.90 ± 0.68 × 10^9^/L, *P* < 0.0001), higher NLR (0.99 ± 0.72 vs. 0.67 ± 0.54, *P* < 0.0001), and lower LNR (0.46 ± 0.26 vs. 0.58 ± 0.25, *P* < 0.0001). Additionally, non-survivors had higher creatinine (0.90 ± 0.47 vs. 0.84 ± 0.34 mg/dL, *P* < 0.0001), higher glucose (118.39 ± 38.66 vs. 108.92 ± 28.02 mg/dL, *P* < 0.0001), higher total bilirubin (median [IQR]: 0.70 [0.50, 1.00] vs. 0.70 [0.50, 0.80] mg/dL, *P* < 0.01), higher rates of ARB use (7.98 % vs. 4.18 %, *P* < 0.0001), CKD (2.45 % vs. 0.87 %, *P* < 0.0001), renal insufficiency (1.88 % vs. 0.43 %, *P* < 0.0001), and high ASA risk (14.31 % vs. 6.99 %, *P* < 0.0001) compared with survivors (Table 1).

**Table 1:** Baseline characteristics of the study population.

Variable	Total (*n* = 7,165)	Survivors (*n* = 5,411)	Non-survivors (*n* = 1,754)	*P* value
Sex, n (%)				<0.0001
Female	2,818 (39.33)	2,218 (40.99)	600 (34.21)	
Male	4,347 (60.67)	3,193 (59.01)	1,154 (65.79)	
Age (years), mean ± SD	59.83 ± 13.62	57.82 ± 13.60	66.05 ± 11.69	<0.0001
BMI, mean ± SD	23.26 ± 3.39	23.64 ± 3.32	22.06 ± 3.36	<0.0001
WBC,10^9^/L, mean ± SD	6.45 ± 2.25	6.33 ± 2.07	6.79 ± 2.70	<0.0001
Hemoglobin, g/dL, median (IQR)	13.20 (11.60, 14.20)	13.20 (11.90, 14.20)	12.20 (10.70, 13.70)	<0.0001
Neutrophil Count,10^9^/L, mean ± SD	3.91 ± 1.95	3.75 ± 1.74	4.40 ± 2.43	<0.0001
Lymphocyte Count,10^9^/L, mean ± SD	1.83 ± 0.70	1.90 ± 0.68	1.62 ± 0.71	<0.0001
NLR	0.75 ± 0.60	0.67 ± 0.54	0.99 ± 0.72	<0.0001
LNR	0.55 ± 0.26	0.58 ± 0.25	0.46 ± 0.26	<0.0001
Creatinine, mg/dL, mean ± SD	0.86 ± 0.37	0.84 ± 0.34	0.90 ± 0.47	<0.0001
Glucose, mg/dL, mean ± SD	111.24 ± 31.23	108.92 ± 28.02	118.39 ± 38.66	<0.0001
Albumin, g/dl, mean ± SD	3.98 ± 0.48	4.06 ± 0.43	3.72 ± 0.51	<0.0001
Total protein, g/dL, mean ± SD	6.84 ± 0.61	6.90 ± 0.58	6.65 ± 0.68	<0.0001
Total bilirubin, mg/dL, median (IQR)	0.70 (0.50, 0.80)	0.70 (0.50, 0.80)	0.70 (0.50, 1.00)	<0.01
ACEI, n (%)				0.05
No	7,127 (99.47)	5,388 (99.57)	1,739 (99.14)	
Yes	38 (0.53)	23 (0.43)	15 (0.86)	
ARB, n (%)				<0.0001
No	6,799 (94.89)	5,185 (95.82)	1,614 (92.02)	
Yes	366 (5.11)	226 (4.18)	140 (7.98)	
β-Blocker, n (%)				0.06
No	6,636 (92.62)	5,030 (92.96)	1,606 (91.56)	
Yes	529 (7.38)	381 (7.04)	148 (8.44)	
Hypertension, n (%)				0.68
No	6,736 (94.01)	5,083 (93.94)	1,653 (94.24)	
Yes	429 (5.99)	328 (6.06)	101 (5.76)	
Diabetes, n (%)				0.87
No	6,975 (97.35)	5,269 (97.38)	1,706 (97.26)	
Yes	190 (2.65)	142 (2.62)	48 (2.74)	
CKD, n (%)				<0.0001
No	7,075 (98.74)	5,364 (99.13)	1,711 (97.55)	
Yes	90 (1.26)	47 (0.87)	43 (2.45)	
Renal insufficiency, n (%)				<0.0001
No	7,109 (99.22)	5,388 (99.57)	1,721 (98.12)	
Yes	56 (0.78)	23 (0.43)	33 (1.88)	
Liver insufficiency, n (%)				0.76
No	7,082 (98.84)	5,350 (98.87)	1,732 (98.75)	
Yes	83 (1.16)	61 (1.13)	22 (1.25)	
ASA, n (%)				<0.0001
Low-risk	6,536 (91.22)	5,033 (93.01)	1,503 (85.69)	
High-risk	629 (8.78)	378 (6.99)	251 (14.31)	
Operative time, min, median (IQR)	210.00 (130.00, 290.00)	210.00 (130.00, 285.00)	220.00 (140.00, 300.00)	<0.0001
Body temperature, °C, mean ± SD	34.73 ± 2.32	34.72 ± 2.37	34.76 ± 2.18	0.45
Heart rate, number, mean ± SD	72.49 ± 11.64	71.19 ± 11.04	76.51 ± 12.47	<0.0001
Diastolic blood pressure, mmHg, mean ± SD	69.28 ± 9.72	69.36 ± 9.67	69.02 ± 9.89	0.21
Systolic blood pressure, mmHg, mean ± SD	117.11 ± 15.45	117.04 ± 15.10	117.32 ± 16.48	0.53

### 3.2 Association between exposure factors and mortality

In three sequentially adjusted Cox regression models, all four inflammatory markers-lymphocyte count, neutrophil count, the lymphocyte-to-neutrophil ratio (LNR), and the neutrophil-to-lymphocyte ratio (NLR)-demonstrated consistent and statistically significant associations with all-cause mortality. The effect sizes gradually attenuated with increasing covariate adjustment.

Specifically, lymphocyte count showed a strong inverse association in the minimally adjusted Model 1 (HR = 0.83, 95 % CI: 0.78–0.89, *P* < 0.0001). This association remained significant in the partially adjusted Model 2 (HR = 0.85, 95 % CI: 0.79–0.92, *P* < 0.0001) and was attenuated, though marginally significant, in the fully adjusted Model 3 (HR = 0.93, 95 % CI: 0.86–1.00, *P* = 0.05). Neutrophil count exhibited a significant positive association in Model 1 (HR = 1.06, 95 % CI: 1.04–1.08, *P* < 0.0001), with an unchanged effect size in Model 2 (HR = 1.06, 95 % CI: 1.04–1.08, *P* < 0.0001). This association attenuated but remained significant in Model 3 (HR = 1.03, 95 % CI: 1.01–1.05, *P* = 0.001). Among all markers, LNR presented the strongest inverse association in Model 1 (HR = 0.51, 95 % CI: 0.42–0.62, *P* < 0.0001), showed minimal change in Model 2 (HR = 0.52, 95 % CI: 0.43–0.62, *P* < 0.0001), and remained highly significant after full adjustment in Model 3 (HR = 0.67, 95 % CI: 0.55–0.82, *P* < 0.0001). NLR had a significant positive association in Model 1 (HR = 1.36, 95 % CI: 1.27–1.45, *P* < 0.0001), showed slight attenuation in Model 2 (HR = 1.34, 95 % CI: 1.25–1.43, *P* < 0.0001), and remained significant in the fully adjusted model (HR = 1.20, 95 % CI: 1.11–1.30, *P* < 0.0001).

Consistent with their inverse mathematical relationship, higher NLR was associated with increased mortality while higher LNR was associated with lower mortality. When analyzed by quartiles (using Q1 as reference), the associations showed varying patterns. For neutrophil count, NLR, and LNR, there were significant monotonic dose-response relationships (*P* for trend < 0.05 for all). For lymphocyte count, although the overall trend was significant (*P* for trend = 0.01), the association was non-monotonic: Q3 (HR = 0.81, 95 % CI: 0.71–0.93, *P* = 0.002) was associated with significantly lower mortality, whereas Q4 (HR = 0.87, 95 % CI: 0.76–1.01, *P* = 0.07) did not reach statistical significance, suggesting a plateau or non-linear pattern. For lymphocyte count, Q3 (HR = 0.81, 95 % CI: 0.71–0.93, *P* = 0.002) was associated with significantly lower mortality, while Q2 (HR = 0.89, 95 % CI: 0.79–1.01, *P* = 0.07) and Q4 (HR = 0.87, 95 % CI: 0.76–1.01, *P* = 0.07) showed non-significant decreasing trends. For neutrophil count, only Q4 (HR = 1.18, 95 % CI: 1.03–1.34, *P* = 0.02) was associated with significantly higher mortality, while Q2 (HR = 0.96, 95 % CI: 0.83–1.11, *P* = 0.59) and Q3 (HR = 1.06, 95 % CI: 0.92–1.22, *P* = 0.45) had no significant associations. For NLR, only Q4 (HR = 1.36, 95 % CI: 1.18–1.56, *P* < 0.0001) was linked to increased mortality, with Q2 (HR = 1.06, 95 % CI: 0.90–1.24, *P* = 0.51) and Q3 (HR = 1.11, 95 % CI: 0.96–1.29, *P* = 0.16) showing no significant effects. For LNR, Q2 (HR = 0.81, 95 % CI: 0.71–0.92, *P* = 0.001), Q3 (HR = 0.77, 95 % CI: 0.68–0.88, *P* < 0.001), and Q4 (HR = 0.76, 95 % CI: 0.65–0.88, *P* < 0.001) were all associated with significantly lower mortality, with similar effect sizes across Q2–Q4, indicating a plateau effect beyond the second quartile (Table 2).

**Table 2:** Association between preoperative inflammatory markers and all-cause mortality (Cox proportional hazards regression analysis).

Variable	Model 1		Model 2		Model 3	
	HR (95 % CI)	*P* value	HR (95 % CI)	*P* value	HR (95 % CI)	*P* value
Lymphocyte count	0.83 (0.78, 0.89)	<0.0001	0.85 (0.79, 0.92)	<0.0001	0.93 (0.86, 1.00)	0.05
Q1	Ref		Ref		Ref	
Q2	0.81 (0.72, 0.92)	<0.001	0.83 (0.73, 0.93)	0.002	0.89 (0.79, 1.01)	0.07
Q3	0.74 (0.65, 0.84)	<0.0001	0.75 (0.66, 0.86)	<0.0001	0.81 (0.71, 0.93)	0.002
Q4	0.76 (0.67, 0.88)	<0.001	0.8 (0.70, 0.92)	0.002	0.87 (0.76, 1.01)	0.07
*P* for trend		<0.0001		<0.0001		0.01
Neutrophil count	1.06 (1.04, 1.08)	<0.0001	1.06 (1.04, 1.08)	<0.0001	1.03 (1.01, 1.05)	0.001
Q1	Ref		Ref		Ref	
Q2	0.91 (0.79, 1.06)	0.22	0.96 (0.83, 1.11)	0.61	0.96 (0.83, 1.11)	0.59
Q3	1.03 (0.90, 1.18)	0.66	1.1 (0.96, 1.27)	0.18	1.06 (0.92, 1.22)	0.45
Q4	1.25 (1.10, 1.41)	<0.001	1.3 (1.14, 1.48)	<0.0001	1.18 (1.03, 1.34)	0.02
*P* for trend		<0.0001		<0.0001		0.01
LNR	0.51 (0.42, 0.62)	<0.0001	0.52 (0.43, 0.62)	<0.0001	0.67 (0.55, 0.82)	<0.0001
Q1	Ref		Ref		Ref	
Q2	0.74 (0.66, 0.84)	<0.0001	0.76 (0.67, 0.85)	<0.0001	0.81 (0.71, 0.92)	0.001
Q3	0.71 (0.62, 0.80)	<0.0001	0.71 (0.63, 0.81)	<0.0001	0.77 (0.68, 0.88)	<0.001
Q4	0.67 (0.58, 0.77)	<0.0001	0.67 (0.58, 0.77)	<0.0001	0.76 (0.65, 0.88)	<0.001
*P* for trend		<0.0001		<0.0001		<0.0001
NLR	1.36 (1.27, 1.45)	<0.0001	1.34 (1.25, 1.43)	<0.0001	1.2 (1.11, 1.30)	<0.0001
Q1	Ref		Ref		Ref	
Q2	1.08 (0.92, 1.27)	0.33	1.11 (0.94, 1.30)	0.22	1.06 (0.90, 1.24)	0.51
Q3	1.14 (0.98, 1.31)	0.08	1.16 (1.01, 1.35)	0.04	1.11 (0.96, 1.29)	0.16
Q4	1.52 (1.33, 1.73)	<0.0001	1.53 (1.33, 1.75)	<0.0001	1.36 (1.18, 1.56)	<0.0001
*P* for trend		<0.0001		<0.0001		<0.0001

### 3.3 Kaplan-Meier survival analysis

Kaplan-Meier (KM) survival curves demonstrated significant differences in survival probabilities across quartiles of all four exposure factors (all log-rank *P* < 0.0001; Figure 2A–D). The *x*-axis represents follow-up time in days, with a maximum follow-up of approximately 4,000 days. For lymphocyte count (Figure 2A), patients in the highest quartile (Q4) exhibited the most favorable survival probability, while those in the lowest quartile (Q1) had the poorest survival; the number at risk for this group declined from 1,778 (Q1) and 1,740 (Q4) at Time 0 to 4 (Q1) and 0 (Q4) by Time 4,000. For neutrophil count (Figure 2B), patients in the highest quartile (Q4) had the worst survival probability, and those in the lowest quartile (Q1) had the best survival; the number at risk decreased from 1,953 (Q1) and 1,963 (Q4) at Time 0 to 24 (Q1) and 0 (Q4) by Time 4,000. For the neutrophil-to-lymphocyte ratio (NLR, Figure 2C), survival probability decreased with increasing quartiles, with Q4 showing the lowest survival rate; the number at risk fell from 1,980 (Q1) and 1,966 (Q4) at Time 0 to 5 (Q1) and 0 (Q4) by Time 4,000. For the lymphocyte-to-neutrophil ratio (LNR, Figure 2D), survival probability increased with increasing quartiles, with Q4 showing the highest survival rate; the number at risk declined from 1,759 (Q1) and 1,759 (Q4) at Time 0 to 3 (Q1) and 29 (Q4) by Time 4,000.

**Figure 2:**
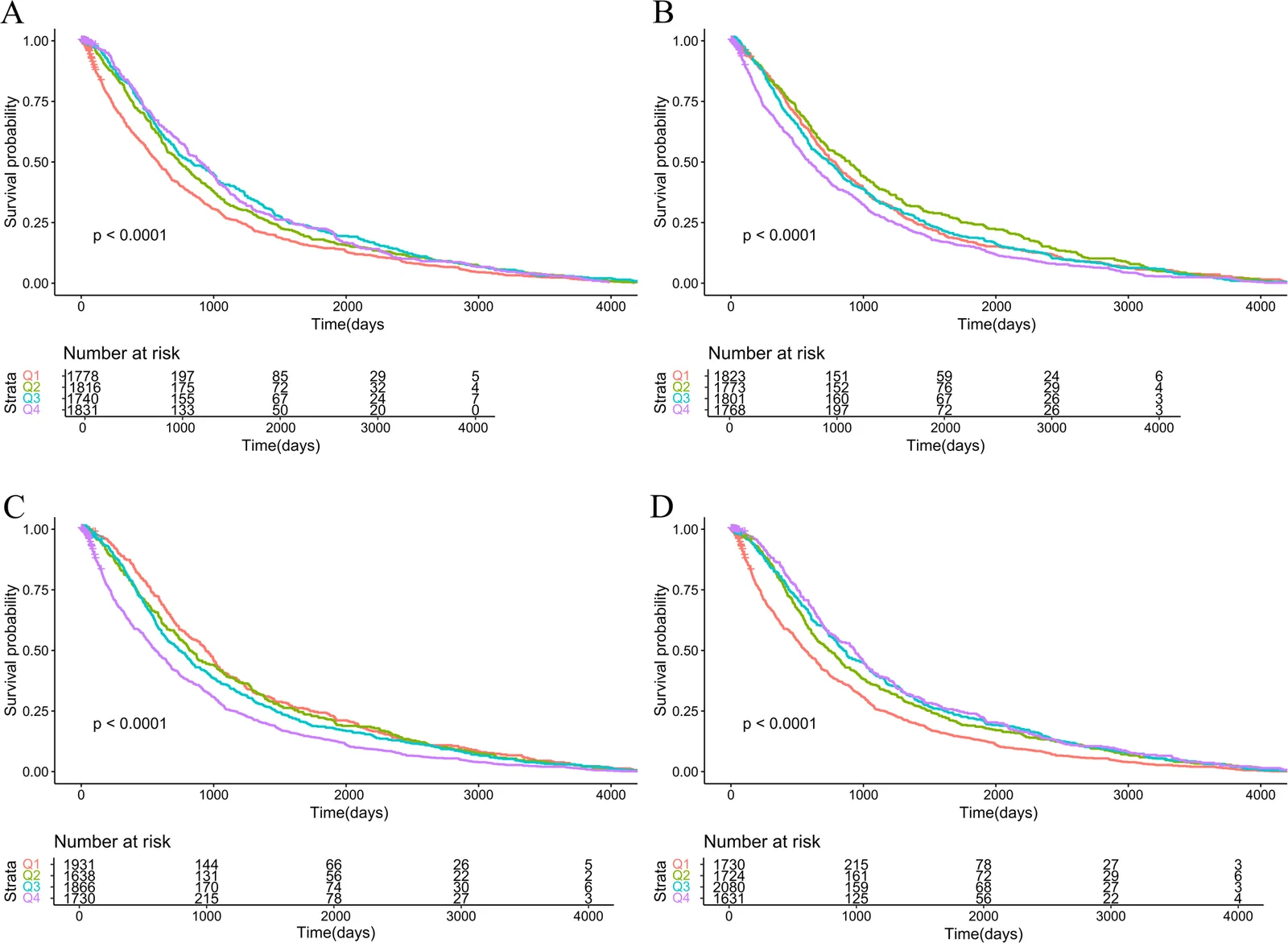
Kaplan-Meier survival curves stratified by quartiles of inflammatory markers. The *x*-axis shows follow-up time in days (maximum approximately 4,000 days). The number at risk at each time point is shown below each panel. (A) Lymphocyte count; (B) neutrophil count; (C) neutrophil-to-lymphocyte ratio (NLR); (D) lymphocyte-to-neutrophil ratio (LNR).

### 3.4 Restricted cubic spline analysis

Restricted cubic spline (RCS) analysis revealed a non-linear relationship between LNR and mortality (*P* for non-linearity = 0.0267). For lymphocyte count, the test for non-linearity was not statistically significant (*P* = 0.0692), indicating that a linear relationship could not be rejected; however, the curve visually suggested a plateauing trend at higher counts (Figure 3B): for LNR (Figure 3A), the mortality risk decreased sharply when LNR was <0.51 (HR = 0.480, 95 % CI: 0.290–0.820, *P* = 0.007) and then remained relatively stable when LNR ≥ 0.51 (HR = 0.748, 95 % CI: 0.481–1.158, *P* = 0.192), while for lymphocyte count (Figure 3B), the mortality risk decreased gradually when lymphocyte count was <1.79 × 10^9^/L (HR = 0.897, 95 % CI: 0.724–1.098, *P* = 0.220) and then plateaued when lymphocyte count ≥1.79 × 10^9^/L (HR = 0.860, 95 % CI: 0.847–1.139, *P* = 0.812). Linear relationships were observed for the neutrophil-to-lymphocyte ratio (NLR) (*P* for non-linearity = 0.6453, Figure 3D) and neutrophil count (*P* for non-linearity = 0.2627, Figure 3C): the mortality risk increased linearly with rising NLR (HR = 1.223, 95 % CI: 0.861–1.738, *P* = 0.261 for <0.67; HR = 1.181, 95 % CI: 1.044–1.300, *P* = 0.007 for ≥0.67) and with rising neutrophil count (HR = 1.021, 95 % CI: 0.965–1.080, *P* = 0.494 for <3.40; HR = 1.019, 95 % CI: 0.983–1.041, *P* = 0.340 for ≥3.40).

**Figure 3:**
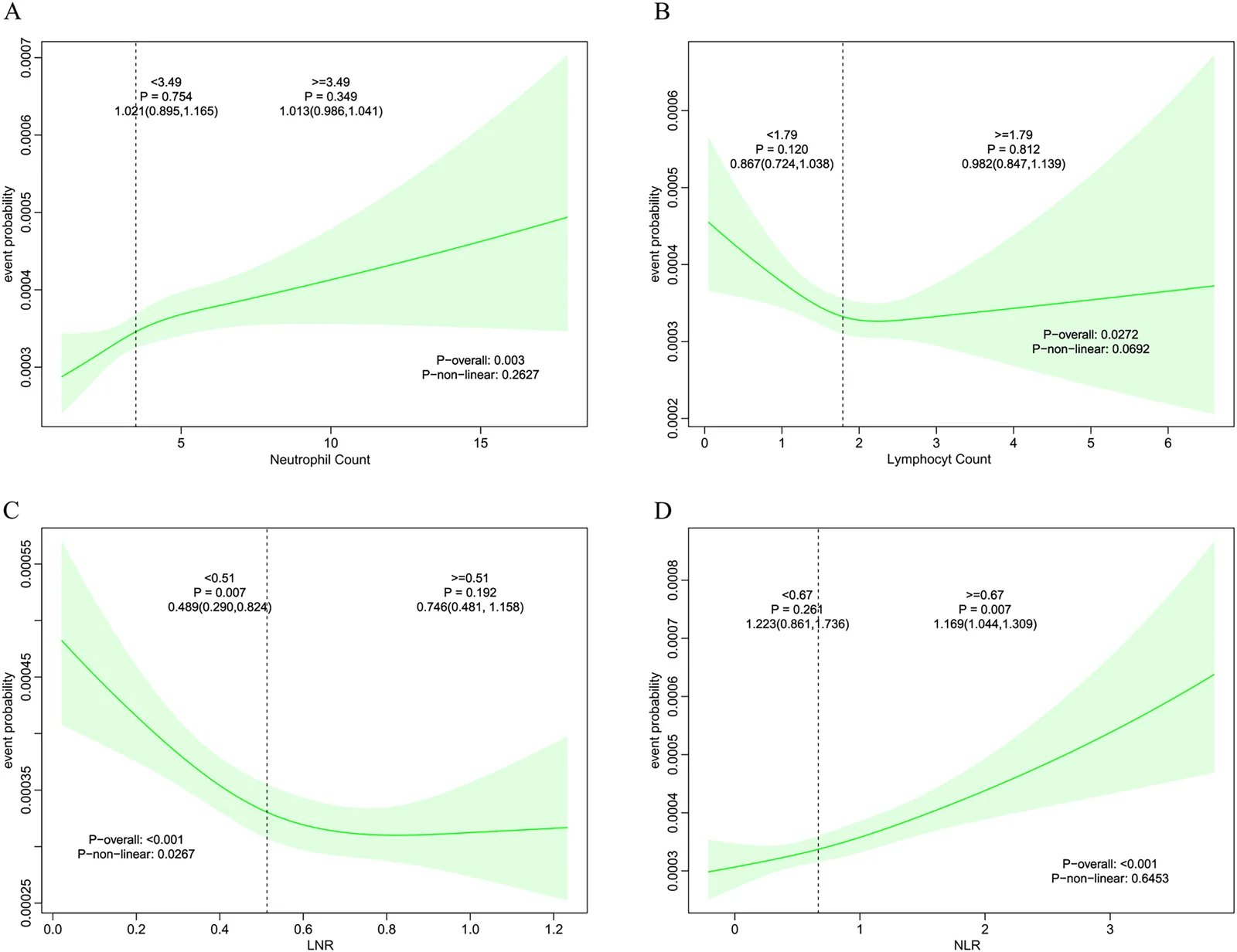
Restricted cubic spline (RCS) plots of the association between inflammatory markers and mortality risk.

### 3.5 Subgroup analysis

Subgroup analyses showed consistent associations between each exposure factor and mortality across sex, age, BMI, hypertension, and diabetes subgroups (Table 3). No significant interaction effects were observed between any exposure factor and subgroup variables (all *P* for interaction > 0.05). For instance, the inverse association between lymphocyte count and mortality was consistent in both female (HR = 0.88, 95 % CI: 0.76–1.01, *P* = 0.07) and male (HR = 0.95, 95 % CI: 0.87–1.04, *P* = 0.29) patients (*P* for interaction = 0.88). Similarly, the positive association between NLR and mortality was consistent across different age groups (<60 years: HR = 1.21, 95 % CI: 1.06–1.39, *P* = 0.004; 60–80 years: HR = 1.16, 95 % CI: 1.03–1.29, *P* = 0.01; >80 years: HR = 1.43, 95 % CI: 1.15–1.79, *P* = 0.002; *P* for interaction = 0.38). For LNR, the inverse association remained significant across BMI subgroups (<18.5: HR = 0.43, 95 % CI: 0.25–0.74, *P* = 0.002; 18.5–24: HR = 0.72, 95 % CI: 0.55–0.94, *P* = 0.02; >24: HR = 0.80, 95 % CI: 0.55–1.17, *P* = 0.24; *P* for interaction = 0.10). For neutrophil count, consistent positive associations were observed in patients with and without hypertension (no hypertension: HR = 1.03, 95 % CI: 1.01–1.05, *P* = 0.003; hypertension: HR = 1.13, 95 % CI: 1.02–1.25, *P* = 0.02; *P* for interaction = 0.47).

**Table 3:** Subgroup analysis of the association between preoperative inflammatory markers and all-cause mortality.

Variable	HR (95 % CI)	*P* value	Pi	Q1	Q2	*P* value	Q3	*P* value	Q4	*P* value	Pi
Lymphocyte count											
Sex			0.88								0.98
Female	0.88 (0.76, 1.01)	0.07		Ref	0.86 (0.69, 1.06)	0.16	0.76 (0.59, 0.98)	0.04	0.85 (0.66, 1.10)	0.21	
Male	0.95 (0.87, 1.04)	0.29		Ref	0.95 (0.81, 1.12)	0.53	0.88 (0.74, 1.04)	0.13	0.97 (0.81, 1.17)	0.74	
Age			0.73								0.39
<60	0.87 (0.76, 0.98)	0.03		Ref	0.80 (0.64, 1.00)	0.05	0.78 (0.62, 0.99)	0.04	0.85 (0.66, 1.09)	0.19	
60–80	0.94 (0.84, 1.06)	0.30		Ref	0.96 (0.8, 1.15)	0.68	0.78 (0.64, 0.96)	0.02	0.94 (0.76, 1.18)	0.61	
>80	0.83 (0.67, 1.02)	0.07		Ref	0.95 (0.69, 1.31)	0.75	1.13 (0.78, 1.66)	0.52	0.73 (0.50, 1.07)	0.10	
BMI			0.12								0.10
<18.5	0.78 (0.61, 1.00)	0.05		Ref	0.80 (0.56, 1.14)	0.22	0.64 (0.44, 0.94)	0.02	1 (0.61, 1.61)	0.98	
18.5–24	0.95 (0.86, 1.05)	0.30		Ref	0.89 (0.75, 1.05)	0.15	0.87 (0.73, 1.05)	0.15	0.87 (0.72, 1.06)	0.16	
>24	0.96 (0.84, 1.09)	0.53		Ref	1.25 (0.96, 1.64)	0.10	1.01 (0.76, 1.34)	0.96	1.14 (0.85, 1.52)	0.39	
Hypertension			0.19								0.72
No	0.94 (0.87, 1.01)	0.11		Ref	0.92 (0.81, 1.05)	0.23	0.85 (0.74, 0.98)	0.02	0.94 (0.81, 1.1)	0.45	
Yes	0.84 (0.53, 1.32)	0.44		Ref	1.19 (0.56, 2.51)	0.65	0.96 (0.46, 2.01)	0.91	1.04 (0.48, 2.28)	0.92	
Diabetes			0.15								0.79
No	0.94 (0.87, 1.02)	0.15		Ref	0.92 (0.81, 1.05)	0.23	0.85 (0.74, 0.98)	0.02	0.94 (0.80, 1.09)	0.38	
Yes	0.19 (0.08, 0.45)	<0.001		Ref	0.13 (0.03, 0.61)	0.01	0.07 (0.01, 1.05)	0.05	0.16 (0.04, 0.71)	0.02	
Neutrophil count											
Sex			0.34								0.00
Female	1.04 (1.00, 1.07)	0.03		Ref	0.87 (0.68, 1.12)	0.28	1.38 (1.09, 1.76)	0.01	1.29 (1.03, 1.60)	0.03	
Male	1.04 (1.01, 1.07)	0.00		Ref	1.04 (0.86, 1.25)	0.72	0.96 (0.80, 1.15)	0.68	1.12 (0.95, 1.33)	0.18	
Age			0.62								0.17
<60	1.04 (1.00, 1.08)	0.04		Ref	0.71 (0.55, 0.92)	0.01	0.76 (0.59, 0.98)	0.04	0.98 (0.78, 1.23)	0.84	
60–80	1.02 (0.99, 1.05)	0.26		Ref	1.02 (0.83, 1.26)	0.86	1.16 (0.94, 1.42)	0.16	1.17 (0.97, 1.41)	0.11	
>80	1.05 (0.99, 1.11)	0.09		Ref	1.06 (0.68, 1.67)	0.79	1.44 (0.93, 2.22)	0.10	1.33 (0.87, 2.01)	0.19	
BMI			0.52								0.72
<18.5	1.01 (0.97, 1.05)	0.68		Ref	1.11 (0.72, 1.72)	0.63	1.29 (0.86, 1.93)	0.21	1.43 (1.01, 2.03)	0.04	
18.5–24	1.03 (1.00, 1.06)	0.03		Ref	0.95 (0.79, 1.16)	0.64	1.04 (0.86, 1.26)	0.66	1.15 (0.96, 1.36)	0.13	
>24	1.08 (1.02, 1.13)	0.01		Ref	0.83 (0.61, 1.13)	0.23	0.88 (0.66, 1.18)	0.40	0.99 (0.75, 1.32)	0.95	
Hypertension			0.52								0.47
No	1.03 (1.01, 1.05)	0.00		Ref	0.99 (0.85, 1.16)	0.94	1.08 (0.93, 1.25)	0.34	1.17 (1.02, 1.35)	0.02	
Yes	1.13 (1.02, 1.25)	0.02		Ref	0.54 (0.24, 1.20)	0.13	1.42 (0.59, 3.42)	0.44	1.86 (0.94, 3.71)	0.08	
Diabetes			0.87								0.90
No	1.03 (1.01, 1.05)	0.00		Ref	0.96 (0.83, 1.11)	0.59	1.07 (0.92, 1.24)	0.37	1.17 (1.02, 1.34)	0.02	
Yes	0.95 (0.70, 1.27)	0.71		Ref	0.71 (0.11, 4.57)	0.72	0.85 (0.21, 3.44)	0.82	0.56 (0.17, 1.82)	0.34	
LNR											
Sex			0.50								0.63
Female	0.54 (0.38, 0.76)	<0.001		Ref	0.78 (0.63, 0.98)	0.03	0.68 (0.54, 0.85)	<0.001	0.68 (0.53, 0.88)	0.00	
Male	0.76 (0.59, 0.98)	0.03		Ref	0.84 (0.72, 0.98)	0.03	0.87 (0.73, 1.03)	0.10	0.85 (0.7, 1.02)	0.08	
Age			0.55								0.47
<60	0.67 (0.47, 0.95)	0.02		Ref	0.81 (0.65, 1.01)	0.06	0.77 (0.61, 0.97)	0.03	0.82 (0.63, 1.05)	0.12	
60–80	0.73 (0.55, 0.96)	0.03		Ref	0.86 (0.71, 1.03)	0.10	0.92 (0.76, 1.12)	0.40	0.77 (0.63, 0.95)	0.01	
>80	0.47 (0.24, 0.89)	0.02		Ref	0.75 (0.55, 1.01)	0.06	0.61 (0.43, 0.86)	0.01	0.94 (0.58, 1.53)	0.81	
BMI			0.10								0.27
<18.5	0.43 (0.25, 0.74)	0.00		Ref	0.83 (0.58, 1.18)	0.29	0.65 (0.45, 0.95)	0.02	0.56 (0.36, 0.87)	0.01	
18.5–24	0.72 (0.55, 0.94)	0.02		Ref	0.80 (0.68, 0.94)	0.01	0.82 (0.69, 0.98)	0.03	0.81 (0.66, 0.98)	0.03	
>24	0.80 (0.55, 1.17)	0.24		Ref	1.01 (0.77, 1.32)	0.95	0.90 (0.67, 1.2)	0.47	0.93 (0.7, 1.24)	0.61	
Hypertension			0.42								0.27
No	0.69 (0.56, 0.85)	<0.001		Ref	0.83 (0.73, 0.94)	0.00	0.83 (0.72, 0.95)	0.01	0.79 (0.68, 0.92)	0.00	
Yes	0.30 (0.11, 0.85)	0.02		Ref	0.69 (0.37, 1.28)	0.24	0.35 (0.17, 0.74)	0.01	0.48 (0.23, 0.99)	0.05	
Diabetes			0.27								0.82
No	0.69 (0.56, 0.84)	<0.001		Ref	0.83 (0.73, 0.95)	0.01	0.81 (0.71, 0.93)	0.00	0.79 (0.68, 0.92)	0.00	
Yes	0.01 (0.00, 0.28)	0.01		Ref	0.38 (0.13, 1.11)	0.08	0.40 (0.12, 1.33)	0.14	0.23 (0.01, 4.68)	0.34	
NLR											
Sex			0.96								0.62
Female	1.32 (1.16, 1.51)	<0.0001		Ref	1.08 (0.82, 1.43)	0.57	1.21 (0.95, 1.56)	0.13	1.55 (1.22, 1.97)	<0.001	
Male	1.17 (1.06, 1.29)	0.00		Ref	1.03 (0.84, 1.26)	0.78	1.01 (0.84, 1.22)	0.90	1.19 (1, 1.43)	0.05	
Age			0.38								0.67
<60	1.21 (1.06, 1.39)	0.00		Ref	0.92 (0.70, 1.22)	0.58	1.03 (0.8, 1.33)	0.80	1.25 (0.98, 1.59)	0.07	
60–80	1.16 (1.03, 1.29)	0.01		Ref	1.23 (0.98, 1.54)	0.08	1.13 (0.92, 1.39)	0.23	1.31 (1.07, 1.6)	0.01	
>80	1.43 (1.15, 1.79)	0.00		Ref	0.86 (0.53, 1.41)	0.56	1.00 (0.64, 1.58)	0.99	1.35 (0.87, 2.08)	0.18	
BMI			0.18								0.26
<18.5	1.32 (1.11, 1.56)	0.00		Ref	1.17 (0.74, 1.85)	0.52	1.44 (0.92, 2.27)	0.11	1.75 (1.17, 2.61)	0.01	
18.5–24	1.16 (1.04, 1.28)	0.01		Ref	1.09 (0.88, 1.36)	0.41	1.04 (0.85, 1.26)	0.71	1.29 (1.07, 1.56)	0.01	
>24	1.15 (0.96, 1.38)	0.13		Ref	0.95 (0.70, 1.29)	0.74	1.13 (0.86, 1.48)	0.37	1.09 (0.82, 1.45)	0.53	
Hypertension			0.29								0.25
No	1.19 (1.10, 1.29)	<0.0001		Ref	1.08 (0.92, 1.28)	0.36	1.09 (0.93, 1.26)	0.29	1.3 (1.12, 1.5)	<0.001	
Yes	1.66 (1.10, 2.51)	0.02		Ref	0.69 (0.29, 1.67)	0.42	1.45 (0.70, 2.99)	0.32	2.09 (1.01, 4.32)	0.05	
Diabetes			0.39								0.83
No	1.19 (1.10, 1.29)	<0.0001		Ref	1.06 (0.90, 1.25)	0.50	1.09 (0.94, 1.26)	0.25	1.3 (1.12, 1.5)	<0.001	
Yes	4.66 (1.62, 13.42)	0.00		Ref	1.78 (0.08, 37.59)	0.71	1.66 (0.08, 35.35)	0.75	4.42 (0.21, 91.29)	0.34	

Pi: P for interaction.

## 4 Discussion

This retrospective cohort study utilizing the INSPIRE database demonstrated that preoperative neutrophil count, lymphocyte count, NLR, and LNR are independent predictors of all-cause mortality in patients undergoing abdominal surgery. The key findings of this study are as follows: (1) Preoperative lymphocyte count and LNR are inversely associated with mortality, while neutrophil count and NLR are positively associated with mortality; (2) Significant dose-response relationships exist for neutrophil count, NLR, and LNR, while lymphocyte count showed a non-monotonic association; (3) A non-linear relationship was observed for LNR, while the relationship for lymphocyte count did not reach statistical significance for non-linearity (*P* = 0.0692), suggesting a predominantly linear or plateauing pattern; linear relationships were noted for NLR and neutrophil count; (4) The associations are consistent across different subgroups, indicating good stability. Although NLR and LNR convey mathematically equivalent information, we present both to accommodate diverse literature preferences and to provide intuitive clinical interpretations on different scales.

Inflammatory responses are critical during the perioperative period, as surgical trauma induces a systemic inflammatory response syndrome (SIRS) characterized by elevated neutrophil counts and reduced lymphocyte counts. Neutrophils are the primary effector cells of the innate immune system, and their excessive activation can lead to tissue damage via the release of reactive oxygen species and proteases. Lymphocytes, in contrast, play a key role in adaptive immunity and anti-inflammatory responses, and their depletion is associated with impaired immune function and increased susceptibility to infections and sepsis. Our findings align with previous studies showing that elevated neutrophil count and decreased lymphocyte count are linked to poor postoperative outcomes [14].

NLR and LNR, as composite markers of inflammation and immune function, have been extensively studied as prognostic biomarkers in various clinical settings. NLR reflects the balance between pro-inflammatory (neutrophils) and anti-inflammatory (lymphocytes) responses, and an elevated NLR indicates a pro-inflammatory state [15]. Previous research has shown that elevated NLR is associated with increased mortality in patients undergoing cancer surgery, cardiac surgery, and critically ill patients. LNR [16], 17], the inverse of NLR, has also been proposed as a prognostic marker, with lower LNR indicating a more severe inflammatory state. Our study confirms the prognostic value of NLR and LNR in abdominal surgery patients, with elevated NLR and decreased LNR being associated with higher mortality.

Although the test for non-linearity for lymphocyte count did not reach statistical significance (*P* = 0.0692), the restricted cubic spline curve suggested a pattern of decreasing risk up to approximately 1.79 × 10^9^/L with a subsequent plateau. The dose-response relationships identified in this study provide valuable clinical insights. For lymphocyte count and LNR, the risk of mortality decreases sharply when these markers are below certain thresholds (1.79 × 10^9^/L for lymphocyte count and 0.51 for LNR), and then plateaus, as evidenced by the similar hazard ratios for LNR Q2, Q3, and Q4 in Table 2 [18], 19]. This suggests that maintaining lymphocyte count above 1.79 × 10^9^/L and LNR above 0.51 may be associated with higher survival probability. For NLR and neutrophil count, the risk of mortality increases linearly with increasing values, indicating that even moderate elevations in these markers are associated with worse outcomes [20], 21]. These thresholds may assist clinicians in identifying high-risk patients and implementing targeted interventions, such as immunomodulatory therapy or enhanced postoperative monitoring.

Subgroup analyses showed that the associations between these hematological markers and mortality are consistent across different sex, age, BMI, hypertension, and diabetes subgroups. This indicates that these markers have prognostic value regardless of patient characteristics, making them suitable for widespread clinical application. For example, the positive association between NLR and mortality is consistent in both young and elderly patients, suggesting that NLR can be used for risk stratification in all age groups [22].

This study has several strengths. First, it utilizes a large-scale, publicly available database with detailed clinical and laboratory data, ensuring sufficient statistical power and generalizability. Second, comprehensive adjustment for potential confounders, including demographic characteristics, comorbidities, surgical variables, and medications, minimizes the impact of confounding factors. Third, multiple statistical methods, including Cox regression, KM analysis, RCS analysis, and subgroup analysis, are used to validate the findings, ensuring robustness. Fourth, the use of routine CBC-derived markers makes the findings applicable in clinical practice, as these tests are widely available and cost-effective.

However, this study also has some limitations. First, as a retrospective cohort study, it cannot establish a causal relationship between exposure factors and mortality. Second, the INSPIRE database is a single-center database, which may limit the generalizability of the findings to other populations. Third, some potential confounders, such as smoking status, alcohol consumption, and nutritional status, are not available in the database and cannot be adjusted for. Fourth, detailed information on surgical indication (e.g., elective vs. emergency) and procedure-specific risk (e.g., contamination class) was not available. While we adjusted for ASA classification, operative time, and hemodynamic variables as proxies for operative severity, residual confounding from unmeasured surgical factors cannot be excluded. Fifth, the follow-up duration varies among patients, which may introduce bias. Sixth, the study does not explore the mechanism underlying the association between these hematological markers and mortality, which requires further experimental studies. Seventh, as the primary objective was to evaluate preoperative markers for preoperative risk stratification, we did not analyze postoperative dynamic changes in these biomarkers. Eighth, approximately 45 % of patients in the INSPIRE database had missing data and were excluded from the analysis. Although missingness was determined to be completely at random and multiple robustness checks (sequential adjustment models, subgroup analyses, etc.) yielded consistent findings, we cannot entirely exclude the possibility of residual selection bias. Future external validation in independent cohorts is warranted.

Future prospective studies that incorporate surgical indication, procedure type, and serial postoperative measurements of the neutrophil-lymphocyte axis are warranted to further elucidate the prognostic role of these markers and to guide dynamic perioperative risk assessment.

## 5 Conclusions

Preoperative neutrophil count, lymphocyte count, NLR, and LNR are independent predictors of all-cause mortality in patients undergoing abdominal surgery. These simple and accessible hematological markers can be used for preoperative risk stratification and may guide perioperative management decisions. Future prospective studies are needed to validate these findings and explore the underlying mechanisms.

## Supplementary Material

Supplementary Material
