# Supplementary material for: Preoperative neutrophil-lymphocyte axis and postoperative mortality: an INSPIRE analysis
Source: Open Life Sci. 2026 Oct 30;21(1):20251378. doi: 10.1515/biol-2025-1378 (PMC13718991; doi:10.1515/biol-2025-1378)
Supplement: Supplementary file 1 [file j_biol-2025-1378_suppl_001.docx]

**Supplementary Table S1:** ICD-10-PCS procedure codes used to define abdominal surgery

| **Code** | **Code** | **Code** |
| --- | --- | --- |
| 0FY00 | 0DTH0 | 0D1K0 |
| 0DTP0 | 0DTH4 | 0D1N0 |
| 0DTP4 | 0DTJ0 | 0DB40 |
| 0DB64 | 0DTJ4 | 0DB44 |
| 0D160 | 0DTQ4 | 0DBN0 |
| 0FT10 | 0WJG0 | 0DBQ0 |
| 0FB00 | 0WJG4 | 0DBQ4 |
| 0DTF0 | 0WBF0 | 0DBS0 |
| 0DTF4 | 0W1G0 | 07TP0 |
| 0WJG0 | 0W3G0 | 07TP4 |
| 0DB60 | 0WUF0 | 0D1E0 |
| 0DJ00 | 0FBG0 | 0DBE4 |
| 0FB04 | 0DT60 | 0DTG0 |
| 0FT20 | 0DT64 | 0DTG4 |
| 0FT44 | 0DBE0 | — |

**Supplementary Table S2:** Comparison of baseline characteristics between included and excluded patients

| **Variable** | **Included** | **Excluded** |
| --- | --- | --- |
| N | 9052 | 7853 |
| Age(Years), mean±SD | 59.99 (13.38) | 56.46 (14.42) |
| BMI, mean±SD | 23.25 (3.41) | 23.69 (3.51) |
| Creatinine,mg/dL, mean±SD | 0.88 (0.48) | 0.88 (0.50) |
| Glucose,mg/dL, mean±SD | 111.42 (32.07) | 107.93 (28.51) |
| Body Temperature, ℃, mean±SD | 34.74 (2.30) | 34.83 (2.29) |
| Heart rate, number, mean±SD | 72.59 (11.70) | 72.01 (11.63) |
| Diastolic Blood Pressure, mmHg, mean±SD | 69.21 (9.77) | 70.21 (9.74) |
| Systolic Blood Pressure, mmHg, mean±SD | 116.99 (15.58) | 119.62 (16.21) |
| Albumin,g/dl, mean±SD | 3.96 (0.49) | 4.07 (0.47) |
| Total Protein, g/dL, mean±SD | 6.83 (0.62) | 6.97 (0.62) |
| WBC, 10^9^/L, mean±SD | 6.44 (2.28) | 6.57 (2.41) |
| Neutrophil Percent, mean±SD | 59.46 (10.83) | 59.70 (11.36) |
| Lymphocyte Percent, mean±SD | 29.19 (9.93) | 29.59 (10.37) |
| Operative Time, min, median(IQR) | 212.50 [135.00, 290.00] | 135.00 [70.00, 230.00] |
| Total Bilirubin, mg/dL, median(IQR) | 0.70 [0.50, 0.80] | 0.70 [0.50, 0.80] |
| Sex, n(%) | 12.90 [11.60, 14.20] | 13.20 [11.90, 14.20] |
| Female | 3532 (39.0) | 4011 (51.1) |
| Male | 5520 (61.0) | 3842 (48.9) |
| ACEI, (%) |  |  |
| No | 8989 (99.3) | 7786 (99.1) |
| Yes | 63 ( 0.7) | 67 ( 0.9) |
| ARB, n(%) |  |  |
| No | 8457 (93.4) | 7359 (93.7) |
| Yes | 595 ( 6.6) | 494 ( 6.3) |
| β-Blocker, n(%) |  |  |
| No | 8255 (91.2) | 7257 (92.4) |
| Yes | 797 ( 8.8) | 596 ( 7.6) |
| CKD, n(%) |  |  |
| No | 8906 (98.4) | 7721 (98.3) |
| Yes | 146 ( 1.6) | 132 ( 1.7) |
| Diabetes, n(%) |  |  |
| No | 8812 (97.3) | 7670 (97.7) |
| Yes | 240 ( 2.7) | 183 ( 2.3) |
| Hypertension, n(%) |  |  |
| No | 8492 (93.8) | 7340 (93.5) |
| Yes | 560 ( 6.2) | 513 ( 6.5) |
| Renal Insufficiency, n(%) |  |  |
| No | 8947 (98.8) | 6813 (86.8) |
| Yes | 105 ( 1.2) | 103 ( 1.3) |
| NA | 0 ( 0.0) | 937 (11.9) |
| Liver insufficiency, n(%) |  |  |
| No | 8915 (98.5) | 6770 (86.2) |
| Yes | 137 ( 1.5) | 146 ( 1.9) |
| NA | 0 ( 0.0) | 937 (11.9) |

**Supplementary Table S3:** Missing data percentage for each variable

| **Variable** | **Missing Values Number** | **Total Sample Number** | **Missing Values Percent** |
| --- | --- | --- | --- |
| Age | 0 | 16905 | 0 |
| Sex | 0 | 16905 | 0 |
| Albumin | 689 | 16905 | 4.08 |
| Creatinine | 103 | 16905 | 0.61 |
| Glucose | 1438 | 16905 | 8.51 |
| Hemoglobin | 679 | 16905 | 4.02 |
| Lymphocyte | 1426 | 16905 | 8.44 |
| Neutrophil | 1428 | 16905 | 8.45 |
| Total Bilirubin | 715 | 16905 | 4.23 |
| Total Protein | 715 | 16905 | 4.23 |
| WBC | 729 | 16905 | 4.31 |
| Body Temperature | 638 | 16905 | 3.77 |
| Heart rate | 3 | 16905 | 0.02 |
| Diastolic Blood Pressure | 1838 | 16905 | 10.87 |
| Systolic Blood Pressure | 1838 | 16905 | 10.87 |
| ACEI | 0 | 16905 | 0 |
| ARB | 0 | 16905 | 0 |
| β-Blocker | 0 | 16905 | 0 |
| CKD | 0 | 16905 | 0 |
| Diabetes | 0 | 16905 | 0 |
| Hypertension | 0 | 16905 | 0 |
| ASA | 1271 | 16905 | 7.52 |
| Renal Insufficiency | 937 | 16905 | 5.54 |
| Liver insufficiency | 937 | 16905 | 5.54 |
| Operative Time | 29 | 16905 | 0.17 |
| BMI | 0 | 16905 | 0 |

**Supplementary Table S4:** Variance inflation factor (VIF) values for covariates

| **Variable** | **Neutrophil Count** | **Lymphocyte Count** | **NLR** | **LNR** |
| --- | --- | --- | --- | --- |
| Sex | 1.30 | 1.30 | 1.30 | 1.31 |
| Age | 1.12 | 1.11 | 1.12 | 1.12 |
| BMI | 1.11 | 1.11 | 1.10 | 1.10 |
| Hemoglobin | 1.73 | 1.78 | 1.73 | 1.74 |
| Neutrophil Count | 1.15 |  |  |  |
| Lymphocyte Count |  | 1.25 |  |  |
| NLR |  |  | 1.36 |  |
| LNR |  |  |  | 1.19 |
| Creatinine | 1.75 | 1.73 | 1.77 | 1.75 |
| Glucose | 1.08 | 1.08 | 1.09 | 1.08 |
| Albumin | 2.77 | 2.67 | 2.75 | 2.71 |
| Total Protein | 2.05 | 2.07 | 2.07 | 2.04 |
| Total Bilirubin | 1.22 | 1.23 | 1.23 | 1.23 |
| ACEI | 1.03 | 1.03 | 1.04 | 1.04 |
| ARB | 1.09 | 1.10 | 1.09 | 1.09 |
| β-Blocker | 1.13 | 1.12 | 1.12 | 1.12 |
| Hypertension | 1.12 | 1.13 | 1.13 | 1.13 |
| Diabetes | 1.10 | 1.11 | 1.10 | 1.10 |
| CKD | 2.21 | 2.20 | 2.22 | 2.19 |
| Renal Insufficiency | 1.81 | 1.82 | 1.82 | 1.80 |
| Liver insufficiency | 1.09 | 1.10 | 1.09 | 1.09 |
| ASA | 1.20 | 1.18 | 1.20 | 1.18 |
| Operative Time | 1.15 | 1.15 | 1.15 | 1.15 |
| Body Temperature | 1.07 | 1.07 | 1.08 | 1.07 |
| Heart rate | 1.23 | 1.20 | 1.25 | 1.23 |
| Diastolic Blood Pressure | 2.10 | 2.07 | 2.08 | 2.08 |
| Systolic Blood Pressure | 2.00 | 1.97 | 1.98 | 1.98 |

## Supplementary Table S5: Justification for covariate selection

| **Category** | **Variables** | **Justification** |
| --- | --- | --- |
| **Demographic and basic vital indicators** | Age, sex, BMI, heart rate, blood pressure, body temperature | Core indicators of baseline status in abdominal surgery patients; confirmed by multiple perioperative prognostic studies to be significantly associated with postoperative mortality risk; routine baseline covariates in surgical prognostic models |
| **Comorbidities** | CKD, hypertension, diabetes | Highly prevalent underlying diseases in abdominal surgery population; directly affect organ function, surgical tolerance, and long-term survival |
| **Medication history** | ACEI, ARB, β-blockers | Commonly used cardiovascular medications; closely related to perioperative hemodynamics and adverse event risk; indispensable confounders in perioperative prognostic research |
| **Laboratory indicators** | Creatinine, glucose, albumin, total protein, total bilirubin, hemoglobin | Comprehensively reflect preoperative liver and kidney function, nutritional status, anemia, and metabolic levels; preoperative poor nutrition and abnormal organ function are independent risk factors for postoperative mortality |
| **Surgery-related indicators** | Operative time | Indirectly reflects magnitude of surgical trauma and complexity; greater trauma associated with stronger systemic inflammatory response and higher mortality risk |
| **ASA classification** | ASA physical status score | Internationally recognized standard for preoperative physical status assessment; gold standard indicator for perioperative risk stratification |
